# Determining the Role of Employee Engagement in Nurse Retention along with the Mediation of Organizational Culture

**DOI:** 10.3390/healthcare11050760

**Published:** 2023-03-05

**Authors:** Ridhya Goyal, Gurvinder Kaur

**Affiliations:** School of Humanities and Social Sciences, Thapar Institute of Engineering and Technology, Patiala 147001, India

**Keywords:** nurse shortage, labor market, retention, employee engagement, organizational culture, self-determination theory

## Abstract

In today’s unpredictable environment, the rapid emergence of the COVID-19 pandemic has shaken the world and its healthcare infrastructure immensely. As nurses are the building blocks of the healthcare personnel labor market, organizations should develop tactics that aid in their retention. With a solid theoretical foundation in self-determination theory, this study aims to understand the role of employee engagement in keeping nurses in 51 hospitals in the Northern Indian region, along with the mediation of organizational culture through smart PLS. In a complementary mediation relationship with organizational culture, nurse retention is positively correlated with employee engagement.

## 1. Introduction

One of the problems with the world’s healthcare system is nurse turnover [1]. When the healthcare system is struggling due to a shortage of resources in a developing economy such as India, where there is already a small pool of trained personnel in the labor market, a pandemic crisis makes it much more difficult. Since healthcare organizations largely depend on labor, they invest heavily in attracting and retaining diversified as well as skilled labor [2]. As a result, attracting and retaining experts is rapidly becoming a primary focus for all health facilities. The recent pandemic has increased the need for healthcare professionals, and nurses are the majority population in the industry with the most patient interaction time [3]. Due to a nursing shortage, the essence of this labor market, with its distinguishable working population, constituting the presence of both registered and unregistered nurses performing interrelated and complementary work, results in ongoing struggles among workers to define their job roles and responsibilities. Registered nurses are the ones who have acquired the General Nursing and Midwifery (GNM) qualification, awarded after training for three years and six months, while those with the Auxiliary Nursing and Midwifery (ANM) qualification, acquired after training for six months to a year in unlicensed private centers, are known as unregistered nurses [4].

Research on actual organizational and professional nurse turnover is scarce [5]. Most of the nurse retention researches have concentrated on intended turnover rather than actual turnover [6]. The absence of seasoned nurses, i.e., nurses aged 52 years or older practicing at the mentor level [7] affects the delivery and continuity of patient care services, which can lead to an increase in adverse events, a loss of nursing care, and patient death [8]. The WHO estimates that there are substantially fewer nurses than the recommended minimum of three, with just one nurse/midwife for every 559 people in Southeast Asia and 1.7 nurses for every 1000 people in India [9]. This shows how the problem of the Indian healthcare market has grown significantly.

Nursing shortages continue to impede the provision of elevated care delivery, negatively impacting health outcomes and increasing the risk of death [10,11]. A worldwide scarcity of nurses is expected in the coming decade [12]. It demonstrates the significance of keeping nursing staff in the healthcare industry. When employees leave, employers lose not only monetarily, but also in terms of valuable information and experience that takes time to regain. 

The healthcare industry has historically lagged behind in terms of engagement, without realizing that an investment towards engagement leads to a psychological contract between the employee and employers [13]. Numerous studies so far have covered the positive relationship between employee engagement and healthcare workers’ retention [12,14,15,16,17].

In terms of nurses’ intent to stay within the organization, there are studies with a strong association between organizational culture and nurse retention (Dols et al., 2019). According to the nursing literature, organizational culture has a significant impact on nurse turnover intent [12,18,19,20]. To date, no studies have examined the mediational role of organizational culture with employee engagement and nurse retention. The present study not only draws upon previous work in the human resource management field concerning the relationship between employee engagement, organizational culture, and nurse retention, but it also identifies the need for a continuing understanding of nurse turnover and employee engagement [12] and establishing the link between the three variables.

Thus, the objective of this paper is to provide a framework that depicts two aspects. First, the impact of employee engagement on retaining the nursing staff, and second, mediation of the organizational culture between employee engagement and the retention of nurses along with the theoretical foundation of self-determination theory.

## 2. Theoretical Framework and Research Hypothesis

### 2.1. Theoretical Underpinning

Self-determination theory (SDT), which encompasses the relationship with all three variables, serves as the theoretical base for this study. While the vast majority of employee engagement programs lack empirical support for their methodologies, the validity of SDT principles has been shown in hundreds of empirical studies over the past 40 years. SDT’s primary point of focus is the people, and how work environmental factors including culture can promote or inhibit that person’s motivation. It is a method of engagement based on evidence, and is in line with the social shift towards individual growth [21]. SDT is also associated with employee retention [22,23].

### 2.2. Employee Engagement

Employee engagement was referred to by Truss et al. [24] as simply “passion for work.” Gadolin and Andersson [25] identified three key factors—professions, organizational structure, and interpersonal relationships—that affect employees’ willingness to engage in providing high-quality healthcare. They came to the realization that all three factors have a positive impact on employee engagement. According to García and Fernández [26], health organizations should understand how to strengthen the element of engagement and encourage it within nursing units in order to achieve ideal medical outcomes.

### 2.3. Organizational Culture

Divyarajaram [27] asserts that OC is essential for motivating staff to uphold a code of conduct, supporting motivation through acknowledgement, fostering self-satisfaction, and acting as a role model for staff behavior. According to Thokozani et al. [28], OC is a company’s emphasis on its staff members, which generates the guiding principles that govern employees’ behavior. All definitions of culture must include the idea that culture is communicated and learnt, as per Odor, H. O. [29]. Because organizational culture evolves over time, it is critical to accurately comprehend the existing organizational culture in order to establish ways to foster a positive nursing organization culture [30]. The survey for the present study was intended to examine the organizational culture in terms of the practices involved in the medical administration system with the immediate supervisor and the head nurse belonging to the medical staff and not the general administrative staff. 

### 2.4. Retention

Employee retention is essentially the desire an employee has to remain with the company. This intent may be predetermined or the outcome of a variety of situations that the employee has been subjected to over time. Kim and Kim [31] examined the rate of turnover and factors associated with attrition among recently recruited nurses and discovered that hospitals should adopt retention tactics to lower turnover rates. Retention is thus the action taken by a company to persuade employees to stay with them and continue working in the same field of professional tasks of the same type by changing rules or approaches. As per Efendi et al. [32], environmental factors of the healthcare system and health center, as well as individual characteristics, influence nurse retention.

### 2.5. Research Hypothesis

#### 2.5.1. Employee Engagement and Retention

Staff members may become more engaged, contented at work, and devoted to the organization if they feel more empowered to promote and carry out projects from the bottom-up, as per Quek et al. [33]. These make it possible to more effectively manage issues including excessive turnover, a lack of qualified workers, and job retention. According to Steiner et al. [34], highly engaged employees have positive perceptions of their company and associated organizational components. Highly engaged workers are less likely to contemplate leaving their current company. Consequently, we hypothesize that:

**Hypothesis** **(H1).** *Employee engagement is positively related to retention.*

##### Employee Engagement and Organizational Culture

As stated by Kashyap and Chaudhary [35], employers should recognize the value of a workplace culture that fosters the development of dependable relationships, strengthens employee identification, and keeps staff members motivated to go above and beyond their regular duties in order to increase engagement at work. Nekula and Koob [36] examined the relationship between engagement and culture and further reported that an organization’s culture positively impacts employee engagement in the healthcare sector. Thus, we hypothesize that:

**Hypothesis** **(H2).** *Employee engagement is positively related to organizational culture.*

##### Organizational Culture and Retention

According to Arasanmi and Krishna [37], businesses incorporating a favorable work environment are able to maintain their top employees for a long time. If workers believe their prospective employer shares their beliefs, they are more likely to stay with the company. Tsarenko et al. [38] stressed the importance of adopting supportive actions that might help employees feel more inclined to stay with a company. On the basis of this, we hypothesize that:

**Hypothesis** **(H3).** *Organizational culture is positively related to retention.*

##### Organizational Culture as Mediator between Employee Engagement and Nurse Retention

In addition to a direct positive effect on retention, engagement may act as a positive influence on organizational culture, which in turn could also lead to a positive effect on the retention of nurses. High levels of commitment to the job are the cause of the connection between engagement and employee intention to leave [39]. Parent and Lovelace [40] demonstrated that many components of an effective company culture are necessary to increase employee engagement. According to them, a positive workplace supports its employees’ organizational culture, which further adds in the engagement as well as the retention process.

Therefore, based on the preceding discussion, we propose the hypothesis:

**Hypothesis** **(H4).** *Organizational culture mediates the relation between employee engagement and nurse retention.*

## 3. Research Methodology

### 3.1. Design and Sample

To ascertain the effects of the independent (employee engagement), dependent (retention), and mediating (organizational culture) variables, a quantitative survey employing a descriptive and cross-sectional design was conducted. Primary data from National Accredited Board of Hospitals and Healthcare Providers (NABH) hospitals in Northern Indian regions of Punjab, Himachal, and Haryana were gathered, and a sample of nurses was selected for this study. The list of hospitals for the survey was extracted with the help of official NABH website. Because staff involvement plays a more ubiquitous role in hospitals with 100 or more beds that were evaluated for the study, the effectiveness of the research is increased. Using the Yamane formula, where the total population (N) was close to 3000 and the margin of error (e) was 0.05, the sample size was determined. Out of 68 hospitals, a sample of 628 registered nurses from 51 NABH hospitals consented to participate in the study, translating to a response rate of 75%. 

The reason for considering NABH hospitals was the successful certification of accreditation informs patients and other stakeholders that a minimal level has been met. Accreditation is the practice of routinely evaluating hospital performance against recognized quality criteria [41]. This method of quality improvement is based on the assumption that the certification process will improve clinical governance and healthcare quality [42]. The duration of data collection was between the pandemic period of June 2020 and January 2021, where a total of 800 questionnaires were sent online with the help of Google Forms, of which 628 were received, giving a response rate of 78.5%. The majority of participants were single females with less than three years of experience bearing the designation of staff nurse with graduate degree (refer Table 1). 

### 3.2. Ethical Consent

The study was approved by the Institutional Ethical committee. Respondents were assured that their participation was confidential and anonymous. Completing and returning the questionnaire constituted consent to participate. 

### 3.3. Data Analysis/Measures

With a five-point Likert scale (1 being strongly disagree, and 5 being strongly agree), all variables were evaluated using a standardized questionnaire. The popularity of variation-based structural equation modeling (SEM) is rising, and there have been many recent advancements and discussions (e.g., Henseler et al. [43] and Rigdon [44]). Using Smart PLS, data analysis was conducted. A multivariate statistical technique called partial least squares (PLS) modeling, which can infer causal relationships, was employed to evaluate the hypotheses [45]. The measurement model and the structural model are used to understand a PLS model in two stages. First, the reliability (item reliability and internal consistency), validity (convergent validity and discriminant validity). Second, the structural model concentrated on the connections between the exogenous and endogenous variables. Based on the relevance of the path coefficients and R^2^ values, the structural model was evaluated.

Common method bias (CMB) can be an issue in cross-sectional studies measuring constructs through indicators on a similar Likert scale. As this study uses cross-sectional data collected on 5-point Likert scale for measuring the latent variables, full collinearity assessment was conducted to rule out the presence of common method bias. All the inner VIF values were found to be less than 3.3, confirming that the data were free from CMB [46].

## 4. Results

### 4.1. Measurement/Outer Model

Table 2 shows the reliability and validity where Cronbach’s alpha and rho A values (ranging between 0.79 and 0.84) were used to assess reliability. The latent variable’s composite reliability values (ranging from 0.85 to 0.88), meanwhile, were above the threshold value of 0.7 [47], showing homogeneity. As shown in Table 2, every latent construct exhibited sufficient convergence validity, with AVE values ranging from 0.54 to 0.61. Three items from culture (C1, C2, C7) and two from engagement (E1, E3) had to be dropped because of low factor loadings resulting in lower Average Variance Extracted (refer Appendix A). After dropping these items, all the AVE values were found to be above 0.5 and all factor loadings of individual items of the reflective constructs retained were found to be above 0.6 [48]. Therefore, the measurement model predicts convergent validity and is reliable.

Content validity was recognized and the instrument was validated by nursing professionals as well as academicians, and the discriminant validity of measurement model was verified with the Fornell–Larcker criterion and heterotrait–monotrait (HTMT) ratio of correlations between the variables. The Fornell–Larcker criterion compares a construct’s correlation coefficients with other constructs to the square root of its AVE. The AVE of each construct in the model should have a square root that is greater than its correlation coefficients with other constructs [49]. As demonstrated in Table 3, the Fornell–Larcker criterion was determined. A new addition to the methods used in the literature to compute discriminant validity is the HTMT ratio [50]. There is extensive use of this discriminant validity test in PLS-SEM. The significance of HTMT ratios is the test’s criteria. All HTMT ratios must be lower than the suggested minimum threshold of 0.90 [50]. As shown in Table 3, the HTMT criterion was achieved. Hence, the discriminant validity of the variables used in this study was confirmed on the basis of both parameters.

### 4.2. Structural/Inner Model

**Figure 1:**
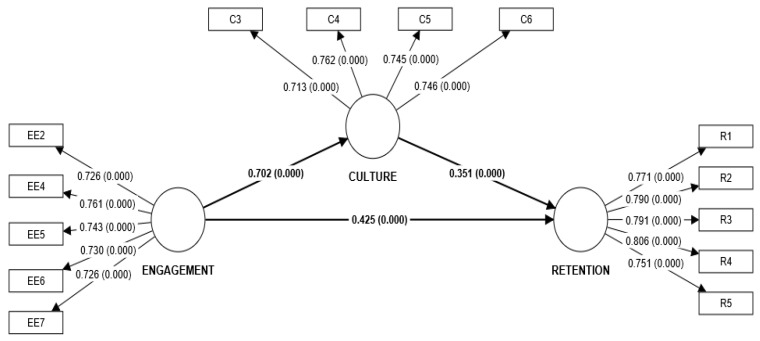
PLS model.

The structural model displays the hypothesized pathways from the research framework. To measure the structural model, the measurements of R^2^, F^2^ (effect size), and Q^2^ were determined (Table 4 and Table 5). These measures examine the predictiveness of the model. The variation explained by endogenous variables is widely used to diagnose structural prediction errors as a multiple correlation coefficient (R^2^) [51]. Becker et al. [52] even advocate for new prediction systems that favor prediction metrics based on R^2^. It is advised to consider values of 0.67, 0.33, and 0.19 as the threshold values for substantial, moderate, and weak predictions, respectively; therefore, the value of R^2^ as per Figure 1 and Table 4 represents moderate prediction, and in Table 5, Q2 values that are greater than zero as a result, show that the exogenous constructs have predictive value for the endogenous construct under consideration [53]. The effect size [54,55,56] is a measure of the magnitude of an effect that is independent of the size of the sample analyzed. The most frequently used is Cohen’s F^2^ coefficient where effect sizes of 0.02, 0.15, and 0.35 are called small, medium, and large [55,56]. Subsequently, the value of F^2^ in Table 4 is somewhat near to medium effect. Furthermore, model fit was calculated through SRMR and its value 0.059, which is below the required value of 0.08, indicating the acceptability of model fit [57].

To evaluate the significance of the relationship, the hypotheses were tested. H1 assesses if the impact of employee engagement on retention is significant. The results revealed that employee engagement has a significant impact on retention. Hence, H1 is supported. H2 evaluates whether employee engagement has a significant impact on culture. The findings show that employee engagement significantly affects culture. Hence, H2 is supported. H3 evaluates whether culture has a significant impact on retention. The results of the study revealed that culture has a substantial impact on retention. Hence, H3 is supported.

### 4.3. Mediation Analysis

As per the result described in Table 6 the direct as well as indirect effects are found to be significant; therefore, we can say that complementary mediation has occurred [58]. The results showed that the inclusion of organizational culture as a mediating variable between employee engagement and retention (β = 0.24, t value = 6.5, *p* value = 0.000) does partially mediate the relationship. Therefore, H4 is partially/complementarily supported.

## 5. Discussion 

Nursing is a profession in a dynamic organization with massive turnover and competence shortages [59]. The significance of the engagement, culture, and turnover intent of nurses, can be explained by looking at why nurses select the nursing profession. According to Guerrero et al. [17], nursing is a career path in which caring is central to the job. Gambino [60] presumed that individuals who join the nursing workforce as a career in order to dedicate themselves to serving others may experience more of a “reality shock,” affecting their desire to remain in the job role. The purpose of this study was to determine the impact of employee engagement on nurse retention along with the mediation of organizational culture with the sound theoretical base of SDT. SDT provides insightful guidance on the instrumental variables comprising of motivational quality and needs satisfaction, along with cultural conditions that can enhance or undermine engagement experiences [21]. This study employs the SDT-based framework with its focus on the three fundamental needs of autonomy, competence, and relatedness and measures employee engagement and organizational culture on these premises. 

This study used PLS-SEM for analyzing the hypothesized relationships. The PLS-SEM technique is recognized for its predictive relevance, and it also does not have any distributional assumptions, which makes it suitable for studies involving behavioral or opinion-based data that do not have multivariate normality [61]. The analysis showed the variable of employee engagement has a positive and significant influence on the variable of nurse retention, which is in agreement with studies such as that by Ekhsan et al. [62]. This finding is critical for the hospitals in India and other countries where the set up in hospitals is not as well organized as other business establishments and the employee engagement activities do not receive adequate attention. Retention of nurses is a prerequisite for having seasoned nurses in the hospital who understand the system and its challenges and can act as experts in guiding the younger nursing staff. Working towards employee engagement of nurses with a more focused approach is expected to yield far-reaching benefits for hospitals as well as society in general. The findings of this research also supported the hypothesis of a complementary mediational role of organizational culture [58] in the relationship between employee engagement and the retention of nurses. This finding is interesting from a theoretical as well as a practical perspective. The role of organizational culture in hospitals has been sparsely studied in the literature. The mediating role of organizational culture in creating an indirect effect on nurse retention with the direct effect of employee engagement provides insights on the dynamics of the process that results in the higher retention of nurses. Thus, the findings of this study suggest that a positive organizational culture reinforces the employee engagement efforts in improving the chances of nurses staying within the organization. As per the foundations of SDT, the building of engagement activities and a culture that supports the employees and motivates them, leads to positive results in terms of affective commitment [63] and talent retention [23]. Hospitals paying attention to the development of a positive culture along with employee engagement activities for the nurses are therefore expected to achieve higher nurse retention. 

This study used cross-sectional data for evaluating the effect of employee engagement activities and organizational culture on retention. As the effect of such activities and the development of culture may take time in manifesting itself, retention can be measured better over a period of time. Further research can be conducted to overcome this limitation of the cross-sectional design by collecting data over a period of time to assess the impact of employee engagement and organizational culture on the retention of nurses in hospitals over time.

## Figures and Tables

**Table 1:** Descriptive statistics.

		Frequency	%	Mean	SD
Gender	Female	421	67		
	Male	207	33		
Age group	21–31	452	72		
	32–42	166	26.4		
	>42	10	1.6		
Marital status	Single	430	68.5		
	Married	198	31.5		
Qualification	Graduate/Diploma	503	80.1		
	Postgraduate	124	19.7		
	Doctorate	1	0.2		
Designation	Staff nurse	461	73.4		
	Supervisor/In charge	148	23.6		
	Head nurse	19	3		
Total experience	<3 years	350	55.7		
	3–6 years	212	33.8		
	6–9 years	53	8.4		
	>9 years	13	2.1		
Income (in rupees, p.a.)	<0.3 million	408	65		
	0.3–0.5 million	199	31.7		
	>0.5 million	21	3.3		
Employee engagement				1.98	0.642
Organizational culture				1.98	0.89
Retention				2.58	0.764

Source: Author’s compilation.

**Table 2:** Reliability and convergent validity.

	Cronbach’s Alpha	rho_A	Composite Reliability	Average Variance Extracted (AVE)
Employee engagement	0.790	0.790	0.856	0.544
Organizational culture	0.728	0.729	0.830	0.550
Retention	0.841	0.842	0.887	0.611

**Table 3:** Discriminant validity.

	Employee Engagement		Organizational Culture		Retention	
	F&L	HTMT	F&L	HTMT	F&L	HTMT
Employee engagement						
	0.737		0.702	0.885		
Organizational culture			0.742			
Retention	0.672	0.824	0.650	0.829	0.782	

**Table 4:** Model explanatory power.

Explanatory Power: R Square	R Square	R Square Adjusted
Retention	0.514	0.513
Organizational culture	0.493	0.493
Effect Size: F Square		
Employee engagement -> retention	0.189	
Organizational Culture -> retention	0.129	
Employee engagement -> org. culture	0.974	

**Table 5:** Model Fit.

	Q2	Model Fit
Culture	0.489	
Retention	0.447	
SRMR		0.059

Note: SRMR = standardized root mean residual.

**Table 6:** Path coefficients of structural model.

DIRECT EFFECTS			
Path	Coefficient	T Statistics	*p*-Values
Employee engagement -> retention	0.426 *	7.025	0.000
Employee engagement -> organizational culture	0.704 *	31.792	0.000
Organizational Culture -> retention	0.352 *	7.971	0.000
INDIRECT EFFECTS			
Path	Coefficient	T Statistics	*p*-Values
Employee engagement -> organizational culture -> retention	0.248 *	6.579	0.000

Note: * shows significant at 1%.

## Data Availability

The datasets generated during and/or analyzed during the current study are available from the corresponding author upon reasonable request.

## Appendix A

**Construct of the Study**	**Item**
EE	EE1 I understand the vision of the hospital.
	EE2 I feel strong & vigorous at my job.
	EE3 I feel enthusiastic about the challenges at work.
	EE4 Hospital conducts engaging activities (for example: games, role play etc.) at regular basis
	EE5 I am satisfied with engaging activities conducted
	EE6 I collaborate with my co-workers to achieve work goals.
	EE7 My team is my inspiration at work
OC	**OC**
	OC1 I can always talk with someone at work if I have work related problem
	OC2 My Seniors treat me with respect.
	OC3 Hospital provides flexible work arrangements.
	OC4 Hospital adapts change quickly.
	OC5 My supervisor recognizes and rewards my effort.
	OC6 My Relationship with colleagues is friendly as well as professional.
Retention	**RTN**
	R1 I see my future in this hospital
	R2 I do not intend to leave the hospital in near future
	R3 Presently, I am not searching for job in another hospital
	R4 It is unlikely that I will look for a job in near future
	R5 I will continue to work in this hospital despite of being offered by other hospital
